# Immunohistochemical characterization of the anti-Müllerian hormone receptor type 2 (AMHR-2) in human testes

**DOI:** 10.1007/s12020-020-02210-x

**Published:** 2020-02-06

**Authors:** A. Sansone, A. M. Isidori, S. Kliesch, S. Schlatt

**Affiliations:** 1 Centre of Reproductive Medicine and Andrology, University Hospital of Münster, Albert-Schweitzer Campus 1, 48149 Münster, Germany; 2 Department of Experimental Medicine, Food Science and Endocrinology, Sapienza University of Rome, 00161 Rome, Italy

**Keywords:** Immunohistochemistry, AMH, AMHR-2, Testis

## Abstract

**Purpose:**

In males, AMH is secreted by immature Sertoli cells; following exposure to endogenous androgens, Sertoli cells undergo a process of maturation which ultimately inhibits AMH expression to undetectable levels in the serum. However, expression of AMH receptor (AMHR-2) has never been studied in human testes, and high intratubular concentrations of AMH have been reported in recent literature. We therefore assessed expression of AMHR-2 in several testicular tissue samples by immunohistochemistry (IHC).

**Methods:**

The IHC method was first validated on tissue samples from healthy human testis (*n* = 2) and from marmoset ovary (*n* = 1). The same method was then used for assessment on testicular histopathology specimens from patients with mixed atrophy (MA, *n* = 2), spermatogenetic arrest (SA, *n* = 2), Sertoli cell-only syndrome (SCO, *n* = 1), Klinefelter syndrome (KS, *n* = 1), and nonseminomatous germ cell tumors (NSGCT, *n* = 1). Tissue samples from two subjects at different pubertal stages (AndroProtect (AP), aged 5 and 14 years) with hematological malignancies were also retrieved.

**Results:**

In adult men, AMHR-2 was expressed on peritubular mesenchymal cells, with patterns closely mirroring α-smooth muscle actin expression. Similar patterns were preserved in almost all conditions; however, in nonseminomatous germ cell tumors the tissue architecture was lost, including AMHR-2 expression. More positive and diffuse staining was observed in tissue samples from prepubertal testes.

**Conclusions:**

In specimens from both healthy and affected testes, AMHR-2 expression appears weaker in adult than in prepubertal tissue sections. The persistence of AMHR-2 expression seemingly hints at a possible effect of intratesticular AMH on the tubular walls.

## Introduction

Anti-Müllerian Hormone (AMH) is a glycoprotein belonging to the TGF-β superfamily [1], produced by granulosa cells of the preantral and small antral follicles in females and by immature Sertoli cells in males [2, 3].

In men, secretion of AMH is largely dependent on the maturity status of the Sertoli cell. In fetal life, expression of AMH by Sertoli cells is triggered by SOX9, later followed by different transcription factors including FSH, SF1, and WT1. Patterns of protein expression change during the first years of life: exposure to endogenous testosterone and morphological changes in Sertoli cells have been reported, including distinct changes in histoskeleton architecture [4, 5].

An ever-growing body of evidence is supporting a role for AMH in the clinical setting, such as the assessment of ovarian reserve for females [6]. However, in males, AMH is mostly used in prepubertal patients: in the fetus, by binding to its receptor [7], AMH induces changes in the morphology of the Müllerian duct mesenchyme, ultimately resulting in apoptosis in the cells of paramesonephric ducts, regression of internal female genitalia, and epithelia-mesenchymal transformation [8]. Mutations in either the protein or the receptor genes impair the binding: male subjects with such alterations develop a rare condition defined persistent Müllerian duct syndrome (PMDS). In PMDS, remnants of the Müllerian ducts are observed in phenotypically normal males [9]. AMH dosage can also prove useful in differential diagnosis between constitutional delay of growth and congenital hypogonadotropic hypogonadism, but little uses are known so far for AMH in adult life.

AMH concentration in serum are significantly reduced after the transition to adulthood; however, significantly higher concentrations are reached in the seminiferous tubules [7], suggesting that AMH secretion might have effects on gonadal health and function even after full testicular maturation. In addition, serum AMH has been investigated as a possible biomarker of spermatogenesis and successful sperm retrieval rate in subjects with nonobstructive azoospermia: while there is limited evidence against the diagnostic value of AMH [10–12], more recent reports suggest that the AMH-to-testosterone ratio could provide useful insight for sperm retrieval techniques [13].

As expected, AMH exerts its biological functions thanks to the interaction with its receptor (AMHR), which is actually a heteromeric complex of types I and II single transmembrane serine/threonine kinase receptors. While the type 2 receptor (AMHR-2) has been identified since the early 1990s [14], the type 1 receptor has been discovered only in more recent times [15, 16]. AMHR-2 seems to be involved in ligand binding, whereas AMHR-1 is a signal transducer shared with other members of the TGF-β superfamily [15]. While some studies have reported different patterns of AMH expression in different testicular affections, such as testicular hypotrophy [17], varicocele [18, 19], and cryptorchidism [20], very little is known concerning how AMHR-2 expression is influenced by gonadal pathologies.

We aimed to assess patterns of AMHR-2 expression both in healthy subjects and in different testicular conditions by performing immunohistochemistry (IHC) on testicular tissue sections. We validated our method in animal models, using marmoset ovary as a positive control, before examining human tissue samples.

## Materials and methods

### Validation protocol

#### Ovarian tissue

Ovarian tissue for validation was obtained from biopsies from healthy adult marmoset (*Callithrix jacchus*) from the institutional breeding colony. Approval for the use of tissue for experimental purposes was provided by LANUV NRW (Az: 84-02.05.50.16014).

#### Testicular tissue

Ethical approval for the use of testicular tissue was obtained from the ethical committee of the Ärztekammer Westfalen-Lippe (no. 2012-555-f-S). Testicular tissue was obtained from testicular samples from the institutional tissue bank consisting of fixed and paraplast-embedded samples. All sections had previously been prepared by using the same protocol: testicular tissue was fixed by immersion in Bouin’s fluid for 24 h, washed in 70% alcohol, dehydrated in a graded series of ethanol, transferred into n-butylacetate as intermedium, and embedded in paraplast before being cut in 5 µm sections. Sections were prepared for staining by deparaffinization and rehydration. Antigen retrieval was performed for 3 min in a microwave oven using citrate buffer as a medium. For validation purposes testes from two men were used who had undergone bilateral orchiectomy for androgen deprivation therapy for prostate cancer. Both samples were selected for qualitatively normal spermatogenesis (according to the Bergmann–Kliesch score [21]).

### Study protocol

#### Testicular tissue

Human testis tissue samples with well-defined pathologies became available from the institutional tissue/data bank (Androbase) in order to assess the expression patterns of AMHR-2 in sections included tissue samples from patients with different testicular affections, including mixed atrophy (MA, *n* = 2), spermatogenetic arrest (SA, *n* = 2), Sertoli cell-only syndrome (SCO, *n* = 1), Klinefelter syndrome (KS, *n* = 1), and nonseminomatous germ cell tumors (NSGCT, *n* = 1). Sections of testicular tissue belonging to two subjects at different stages of puberty (AndroProtect (AP), aged 5 and 14 years) with hematological malignancies were also retrieved.

#### Immunohistochemistry (IHC)

The primary antibody used for this study was a monoclonal mouse antibody against AMHR-2 produced by Abcam, Cambridge, MA, USA (ab64762). According to the product datasheet, the antibody would bind to the aminoacidic sequence NANYSHLPPPGSPG, corresponding to amino-acids 117–130 of Human AMHR-2 and be valid of IHC staining following heat-mediated antigen retrieval.

#### Staining technique

After rehydration and washing, tissue sections were incubated in primary antibody, using different dilutions. For the validation protocol, marmoset ovarian sections were stained for AMHR-2 using three dilutions of the antibody (1:100, 1:200, 1:500), and human testicular sections were stained with six different dilutions (1:100, 1:200, 1:500, 1:1000, 1:5000, 1:10000). For the study protocol, two different dilutions were performed for all tissue sections (1:250, 1:500). Incubation was performed for 2 h for each section. An indirect streptavidin–biotin method (Dako REAL™ Detection System, Alkaline Phosphatase/RED, Rabbit/Mouse, Code K5005) with a Fast Red-type chromogen was used for 20 min. Each incubation step was followed by three washes (5 min each) in buffer (0.05 M Tns, 0.15 M Nacl, pH 7.6). After incubation, all sections were washed with tap water and counter-stained with hematoxylin.

#### Control staining

Specificity of the staining method was tested by serial dilution (as previously described) or omission of the primary antibody. In addition, we also used a different monoclonal mouse antibody, namely against α-smooth muscle actin (α-SMA, 1:500) (Sigma Chemie GmbH, code No. 1-2547) on selected testicular sections from the validation protocol, as a measure of the staining’s validity. The IHC staining pattern associated with this antibody has already been described in literature [22].

#### Histological examination

IHC images were acquired using a PreciPoint^®^ M8 microscope (Precipoint, Freising, Germany) with ×20 and ×60 magnification and recorded using the ViewPoint^®^ software.

## Results

### Validation protocol

**Fig. 1:**
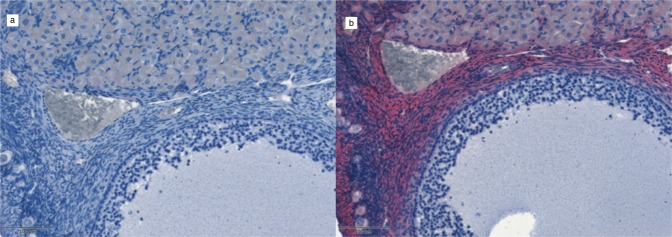
Validation protocol for AMHR-2 IHC staining. Tissue sections from marmoset ovary; ×20 magnification. **a** primary antibody omitted, using hematoxylin counterstaining only; **b** 1:500 AMHR-2 staining and hematoxylin counterstaining

**Fig. 2:**
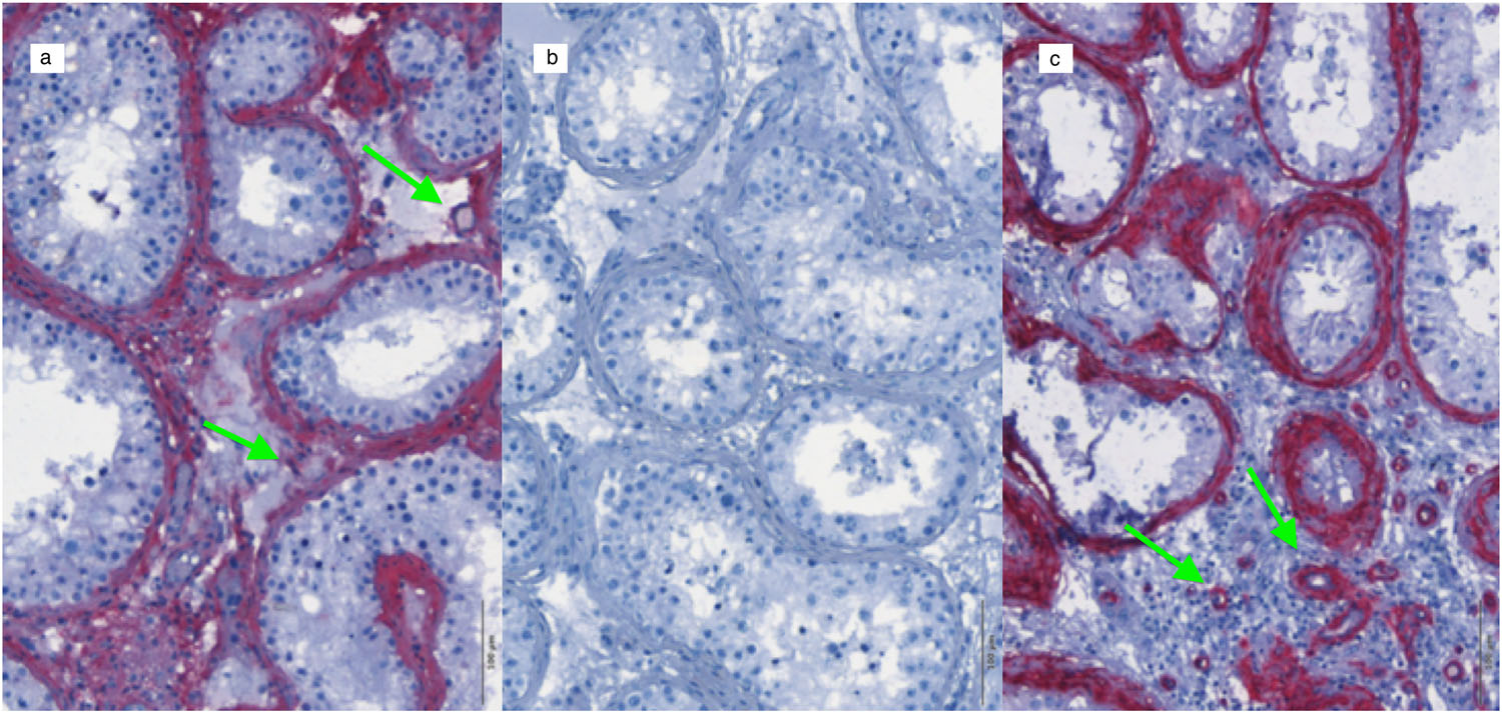
Validation protocol for AMHR-2 IHC staining. Tissue sections from human testis; ×20 magnification. **a** 1:500 AMHR-2 staining and hematoxylin counterstaining; **b** primary antibody omitted, using hematoxylin counterstaining only; **c** 1:500 α-SMA staining and hematoxylin counterstaining. The green arrows point towards blood vessels

Both in the human testicular and marmoset ovarian sections, AMHR-2 positive cells were identified in all titrations of the primary antibody with the same pattern of expression. In marmoset ovary (Fig. 1a, b), AMHR-2 positive cells were identified in the granulosa cell layer surrounding the oocyte; in human testis, mesenchymal peritubular cells resulted markedly stained by the primary antibody, with some resemblance to α-SMA staining (Fig. 2a–c). As highlighted by the arrows in Fig. 2a, c, however, despite the similarities, blood vessels in testis samples are weakly labeled for AMHR-2 and strongly labeled for α-SMA.

### Study protocol

**Fig. 3:**
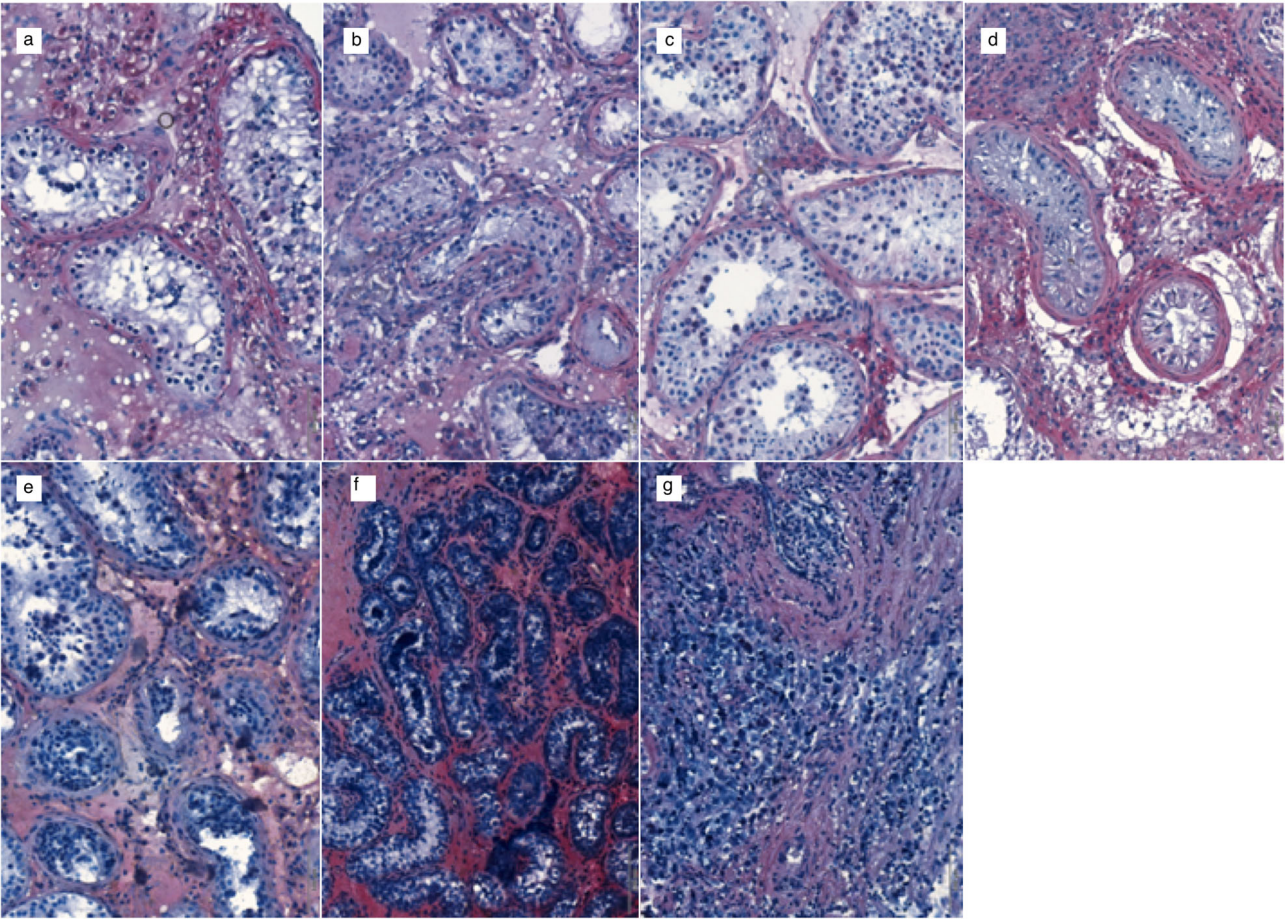
Study protocol for AMHR-2 IHC staining. Tissue sections from human testis; ×20 magnification; 1:500 AMHR-2 staining and hematoxylin counterstaining. **a** Spermatogenetic arrest. **b** Sertoli cell-only syndrome. **c** Mixed atrophy. **d** Klinefelter syndrome. **e** Hematological malignancy (non-Hodgkin lymphoma), 14 years old. **f** Hematological malignancy (Hodgkin lymphoma), 5 years old. **g** Nonseminomatous germ cell tumors

Following the completion of our validation protocol, we performed the same IHC staining on testicular sections from seven patients with different testicular affections (Fig. 3a–g). The same patterns of expression for AMHR-2 were identified for SA (Fig. 3a), SCO (Fig. 3b), MA (Fig. 3c), and KS (Fig. 3d) patients: mesenchymal cells showed markedly positive staining in a thin layer surrounding tubular structures. As previously stated, IHC staining was also performed on two patients with hematological disease belonging to the AP project [23] undergoing cryopreservation of testicular tissue before full testicular maturation. In the tissue sections from a 14-year-old AP patient with non-Hodgkin lymphoma an adult-like pattern in AMHR-2 expression could be identified (Fig. 3e); contrarywise, in the tissue sections from a 5-year-old AP patient with Hodgkin lymphoma, intense, diffuse staining was observed (Fig. 3f) suggesting a change in the expression patterns of AMHR-2 during the transition through puberty. In tissue scans from the NSGCT patient, loss of testicular architecture was observed and AMHR-2 expression was therefore different from all remaining samples.

## Discussion

This is the first study, to our knowledge, providing a detailed IHC characterization of AMHR-2 expression in healthy subjects as well as in a series of different testicular conditions. Our results suggest that expression of AMHR-2 is decreased following the transition to adulthood in all tissue samples, mirroring reduced AMH concentrations: a progressively more diffused, intense staining was observed in prepubertal and pubertal tissue samples than in all other sections. Even in adults, however, AMHR-2 staining showed distinctive features: a thin layer of peritubular mesenchymal cells showed positive staining, suggesting a possible role for AMH even in adult testis. Despite almost nonmeasurable serum levels, intratubular concentrations of AMH are far greater [7, 24]. Several studies have reported a progressive decrease of AMH levels during aging [25–27], suggesting a progressive decline in Sertoli cell function, which is also fitting with current hypothesis on the negative effects of aging on the male reproductive system [28, 29]. As previously mentioned, several authors have investigated the possibility of AMH measurement as a prognostic tool for successful sperm retrieval with testicular sperm extraction techniques (TESE or microTESE): a meta-analysis study conducted by Toulis et al. [10] concluded that while the diagnostic value of AMH is not supported, the evidence in these regards is extremely limited. However, more recently, Alfano et al. [13] reported different results, with both AMH and AMH-to-testosterone ratio achieving with a predictive accuracy of 93 and 95% for sperm retrieval rate via microTESE. In patients with Klinefelter syndrome, no association was found between biomarkers of Sertoli cell function, including AMH, and microTESE success [30]; indeed, the pattern of AMH expression in Klinefelter patients is somehow different from normal, with a delay in the FSH-mediated decline occurring in puberty, possibly due to a temporary, functional compensation of Sertoli cells [31]. Still on the topic of different patterns of expression, Kistamás et al. have not found any statistically relevant correlation between AMHR-2 expression and age on the appendix testis [32] in a large series of patients undergoing surgical explorations for different conditions, such as hydrocele and varicocele; however, as the appendix testis is histologically different from testicular tissue [33], drawing an association with the present study is not feasible.

Little evidence has been found concerning the possible role of AMH in spermatogenesis [24]: a positive association between AMH concentrations (both in serum and in seminal plasma) and sperm quality has been reported in a small number of studies, although others have found no significant association. Peritubular cells are involved in passive motility of sperm cells: at the end of spermatogenesis spermatozoa are immotile, and sperm transit is allowed by rhythmic contractions of such peritubular cells. The similar patterns of expression for AMHR-2 and α-SMA possibly suggest that in fact AMH in adult life could be involved in the regulation of sperm transit through tubules, partly supporting the positive association between sperm parameters and AMH concentrations in seminal plasma reported in some studies [24]. Expression of α-SMA is induced by androgens and FSH [22], which at the same time act as triggers for downregulation of AMH secretion. The persistence of AMHR-2 in adult life might hint at a different physiologic role for AMH after the end of puberty: AMHR-2 is no longer expressed by Sertoli cells, as expected following the end of the maturation process, but is still expressed by peritubular cells which support the passive movement of sperm. Several factors are involved in the regulation of peritubular cell contraction and relaxation [34]: this could perhaps provide an explanation to inconclusive evidence linking AMH levels to sperm parameters.

In our tissue sections, AMHR-2 expression is also preserved in testicular pathologies, such as Klinefelter syndrome and Sertoli cell-only syndrome; this is somewhat in contrast with previous reports suggesting that the tubular wall compartment is remodeled in men with impaired spermatogenesis [34].

## Conclusions

Our IHC study showed that AMHR-2 is expressed in testicular tissue sections, both in humans and in animal models, in a thin layer of peritubular mesenchymal cells. Expression of AMHR-2 is conserved in healthy controls and in different testicular pathologies; patterns of expression are remarkably similar to α-SMA. Expression of AMHR-2 is clearly different before the onset of puberty, closely mirroring serum AMH levels. However, while in adult males serum AMH is almost negligible, the significantly higher concentrations of this hormone in tubular fluid might be enough to elicit a response from the receptor on the peritubular wall, possibly resulting in changes in sperm parameters. However, given the little evidence suggesting a link between sperm parameters and AMH levels, our hypothesis requires further testing.
